# Activation of Akt by SC79 protects myocardiocytes from oxygen and glucose deprivation (OGD)/re-oxygenation

**DOI:** 10.18632/oncotarget.14785

**Published:** 2017-01-21

**Authors:** Koulong Zheng, Qing Zhang, Gang Lin, Yefei Li, Zhenqiang Sheng, Jue Wang, Liang Chen, Hui-he Lu

**Affiliations:** ^1^ Department of Cardiology, The Second Affiliated Hospital of Nantong University, Nantong, China

**Keywords:** ischemic heart diseases, oxygen glucose deprivation (OGD), myocardial cells, SC79, Akt

## Abstract

SC79 is a novel Akt activator. The current study tested its potential effect against oxygen and glucose deprivation (OGD)/re-oxygenation-induced myocardial cell death. We showed that SC79 activated Akt and protected H9c2 myocardial cells and primary murine myocardiocytes from OGD/re-oxygenation. Reversely, Akt inhibitor MK-2206 or Akt1 shRNA knockdown almost completely abolished SC79-mediated myocardial cytoprotection. SC79 treatment in H9c2 cells inhibited OGD/re-oxygenation-induced programmed necrosis pathway, evidenced by mitochondrial depolarization and cyclophilin D-p53-ANT-1 (adenine nucleotide translocator 1) association. Further, SC79 activated Akt downstream NF-E2-related factor 2 (NRF2) signaling to suppress OGD/re-oxygenation-induced reactive oxygen species (ROS) production. Reversely, NRF2 shRNA knockdown in H9c2 cells largely attenuated SC79-induced ROS scavenging ability and cytoprotection against OGD/re-oxygenation. Together, we conclude that activation of Akt by SC79 protects myocardial cells from OGD/re-oxygenation.

## INTRODUCTION

Ischemic heart diseases are major threat to human health, which contribute to significant human mortalities each year [1, 2]. The incidence of these diseases, on the other hand, has been steadily rising [1, 2]. Thus, understanding the associated pathological mechanisms and developing possible intervention strategies are vital to fight these diseases [1, 2]. Our lab [3, 4] and others have been applying oxygen glucose deprivation (OGD) in cultured myocardial cells to mimic ischemic cell damages [5–7]. Sustained OGD (> 1 hour) coupling with re-oxygenation is shown to disrupt mitochondrial function, causing reactive oxygen species (ROS) production and cell necrosis (but not apoptosis) [5–7].

Recent research efforts have developed a specific, potent and cell-permeable Akt small molecule activator, named SC79 [8]. SC79 inhibits Akt membrane translocation, yet simultaneously activates Akt in the cytosol [8]. Existing studies have reported the pro-survival potential of this compound in various experimental settings [8–11]. For example, Jo *et al*., showed that SC79 protects neurons from stroke *in vivo* and *in vivo*. Gong *et al*., recently demonstrated that SC79 activates Akt signaling and protects retinal pigment epithelium cells from UV radiation [11]. Similarly, this novel Akt activator could rescue osteoblasts from dexamethasone [10]. The potential effect and the underlying signaling mechanisms of SC79 against OGD/re-oxygenation-induced myocardial cell death were tested in the current study.

NF-E2-related factor 2 (NRF2) dictates the transcription of key anti-oxidant genes via coupling with antioxidant-responsive element (ARE) in nuclei [12, 13]. Transcription of these genes, including *heme oxygenase-1 (HO1)*, *NADPH quinone oxidoreductase 1 (NQO1)* and *glutamate cysteine ligase catalytic subunit (GCLC)*, could significantly inhibit ROS production and oxidative stress [14]. It is known that ROS production is the primary upstream mechanism to provoke the above mitochondrial necrosis pathway by OGD/re-oxygenation [3, 15]. Indeed, several cytoprotective agents, including salidroside and the SphK1 activator K6PC-5, were shown to inhibit OGD/re-oxygenation-induced ROS production and subsequent mitochondrial necrosis in myocardial cells [3, 15]. In the current study, we show that SC79 protects myocardial cells from OGD/re-oxygenation via activating Akt downstream Nrf2 signaling.

## RESULTS

### SC79 protects myocardial cells from OGD/re-oxygenation

**Figure 1:**
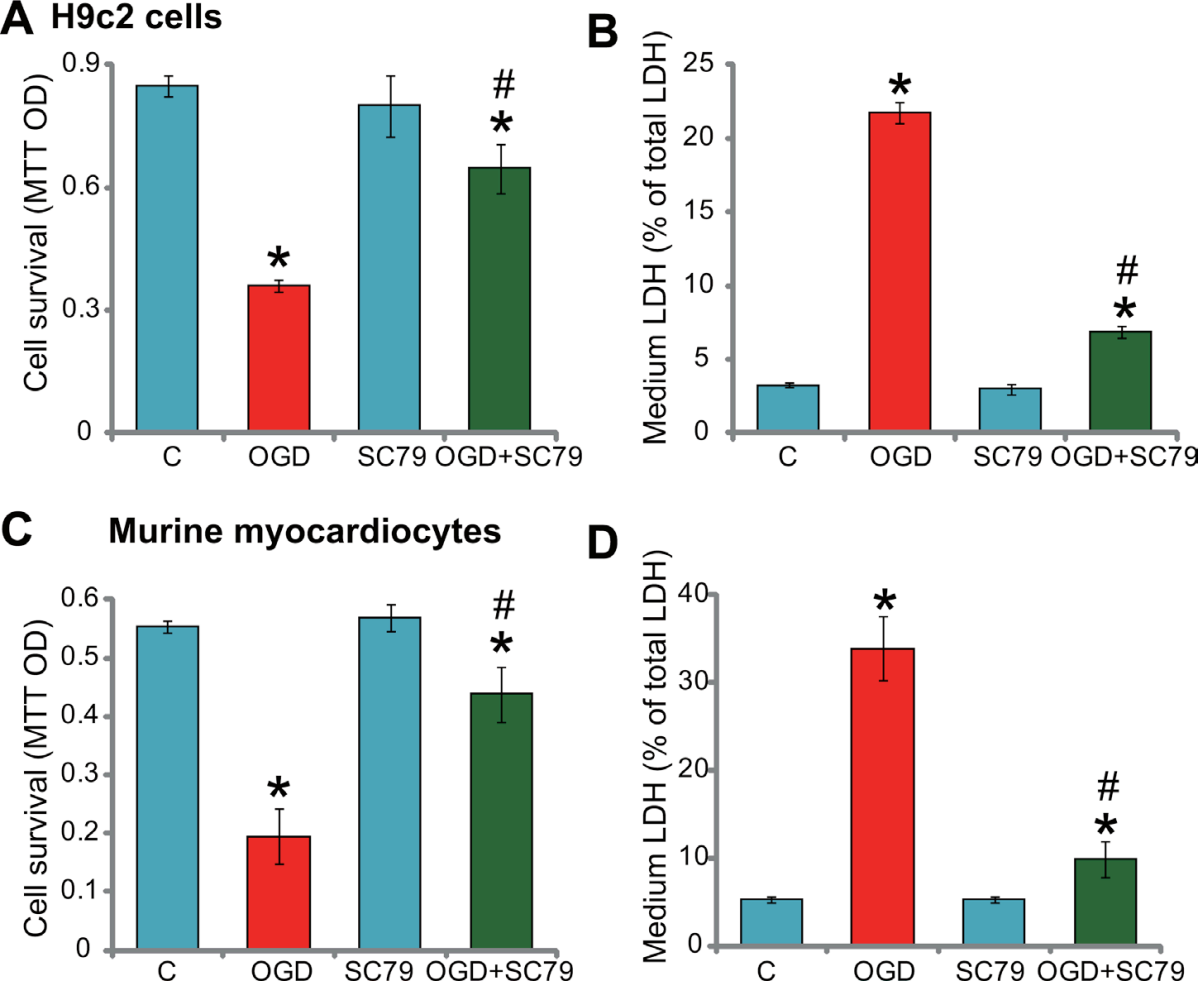
SC79 protects myocardial cells from OGD/re-oxygenation H9c2 myocardial cells (**A** and **B**) or primary murine myocardiocytes (**C** and **D**), pretreated with/out SC79 (10 μM, 1 hour pretreatment), were maintained under OGD for 4 hours, followed by 24 hours of re-oxygenation. Afterwards, cell survival was tested by MTT assay (A and C); Cell death was examined by the LDH release assay (B and D). “C” stands for untreated control (Same for all figures). “OGD” stands for OGD/re-oxygenation (Same for all figures). Bars indicate standard deviation (SD, *n* = 5). Each experiment was repeated five times and similar results were obtained. *p < 0.05 vs. group “C”. ^#^p < 0.05 vs. “OGD” group.

To study the potential effect of SC79 on ischemic damages, H9c2 myocardial cells [3, 4] were maintained under oxygen glucose deprivation (OGD) for 4 hours, which were then subjected to re-oxygenation for additional 24 hours. In line with our previous findings [3, 4], OGD mimicked ischemic damages and significantly inhibited H9c2 cell survival [MTT optic density (OD) reduction, Figure 1A]. Remarkably, pre-treatment for 1 hour with the Akt activator SC79 (10 μM) [8, 9, 11] significantly attenuated OGD/re-oxygenation-induced H9c2 survival reduction (Figure 1A). Meanwhile, results in Figure 1B demonstrated that SC79 pre-treatment also dramatically inhibited OGD/re-oxygenation-induced H9c2 cell death, the latter was tested by increased lactate dehydrogenase (LDH) release in the conditional medium [3, 4].

Next, the primary murine myocardiocytes were cultured (see Method). The above OGD (4 hours)/re-oxygenation (24 hours) treatment again significantly inhibited cell survival (Figure 1C), and provoked cell death (Figure 1D). Similarly, pre-treatment with SC79 again dramatically decreased OGD/re-oxygenation-induced injuries to the primary murine myocardiocytes (Figure 1C and 1D). Thus, SC79 efficiently protects myocardial cells from OGD/re-oxygenation. Notably, treatment with the SC79 by itself had no significant effect on survival and death of above myocardial cells (Figure 1A–1D).

### SC79-induced myocardial cytoprotection against OGD/re-oxygenation requires Akt activation

**Figure 2:**
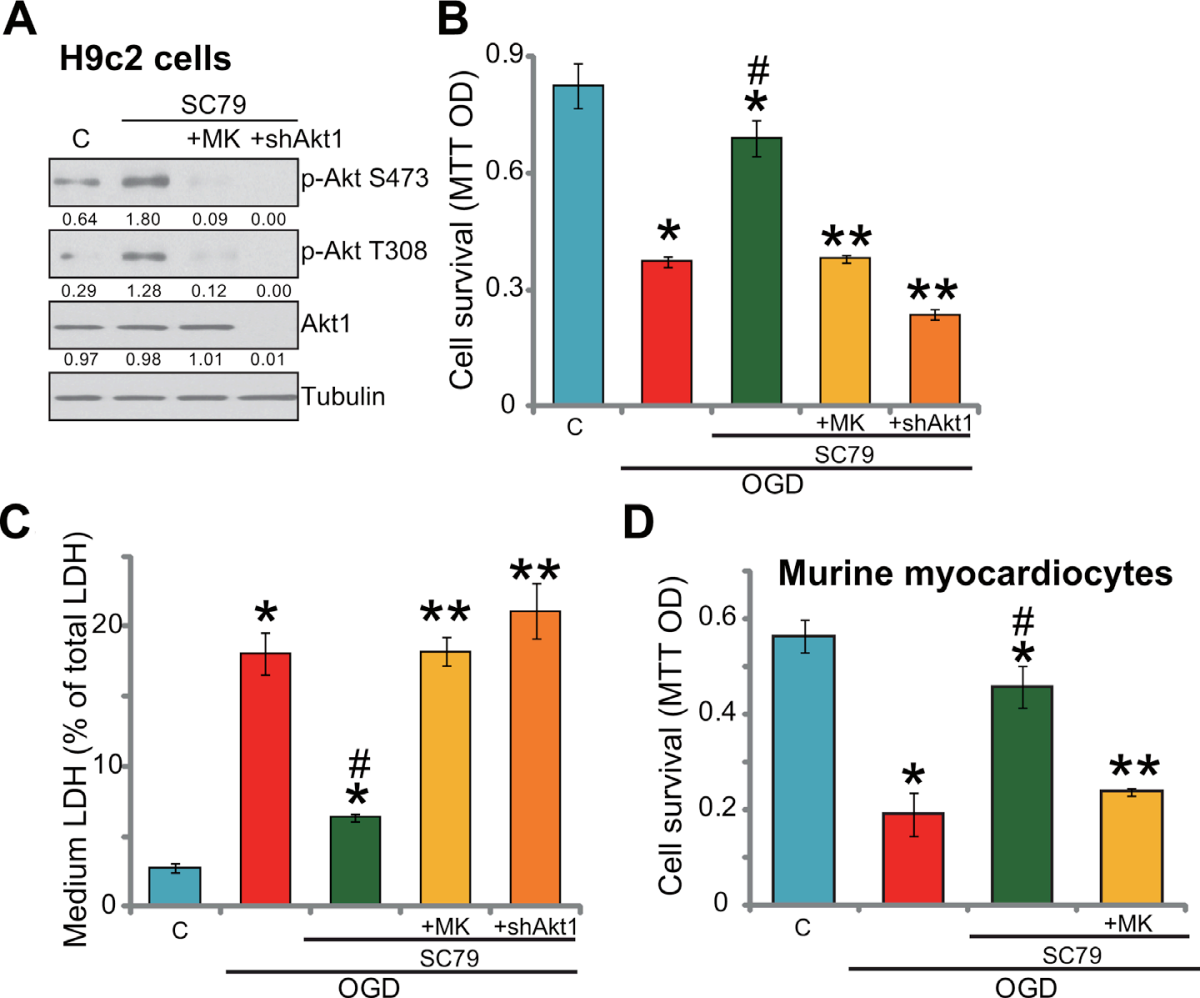
SC79-induced myocardial cytoprotection against OGD/re-oxygenation requires Akt activation H9c2 myocardial cells (**A**–**C**) with/out Akt1 shRNA (“shAkt1”), or the primary murine myocardiocytes (**D**) were treated with SC79 (10 μM) or plus MK-2206 (“MK”, 10 μM) for 1 hour, expression of indicated proteins was shown (A); After the above treatment, cells were further subjected to OGD/re-oxygenation stimulation for 24 hours, cell survival (B and D, MTT assay) and cell death (C, LDH release assay) were tested. Akt1 expression (vs. Tubulin) and Akt phosphorylation (vs. Tubulin) were quantified. Bars indicate standard deviation (SD, *n* = 5). Each experiment was repeated three times and similar results were obtained. *p < 0.05 vs. “C” group. ^#^*p* < 0.05 vs. “OGD” group. ^*^p < 0.05 vs. “SC79” group.

As discussed, SC79 is a newly-developed Akt activator [8, 9, 11, 16]. We thus tested Akt signaling in SC79-treated myocardial cells. Western blot assay results in Figure 2A showed that treatment with SC79 (10 μM, 1 hour) in H9c2 cells induced significant Akt activation, which was tested by phosphorylation (“p-”) of Akt at both Ser-473 and Thr-308 [11, 17–19]. MK-2206, the Akt specific inhibitor [20], almost blocked Akt activation by SC79 (Figure 2A). Significantly, SC79-mediated H9c2 cytoprotection against OGD was largely attenuated with MK-2206 co-treatment (Figure 2B and 2C). In another words, SC79 was almost ineffective against OGD/re-oxygenation in the presence of MK-2206 (Figure 2B and 2C). These results suggest that Akt activation is required for SC79-induced myocardial cytoprotection against OGD/re-oxygenation.

To further support the above hypothesis, shRNA strategy was applied to knockdown Akt1. As demonstrated, the lentiviral Akt1 shRNA led to almost complete depletion of Akt1 in H9c2 cells (Figure 2A). Consequently, SC79-provoked Akt activation was completely blocked in Akt1-silenced cells (Figure 2A). Remarkably, as shown in Figure 2B and 2C, SC79 was similarly ineffective against OGD/re-oxygenation in Akt1-silenced H9c2 cells. Significantly, the Akt specific inhibitor MK2206 almost nullified SC79-mediated cytoprotection in the primary murine myocardiocytes (Figure 2D). These shRNA results further confirmed that Akt activation is required for SC79-indued myocardial cytoprotection against OGD/re-oxygenation.

### OGD/re-oxygenation-induced myocardial cell death is exacerbated with Akt inhibition, but is attenuated with forced-activation of Akt

**Figure 3:**
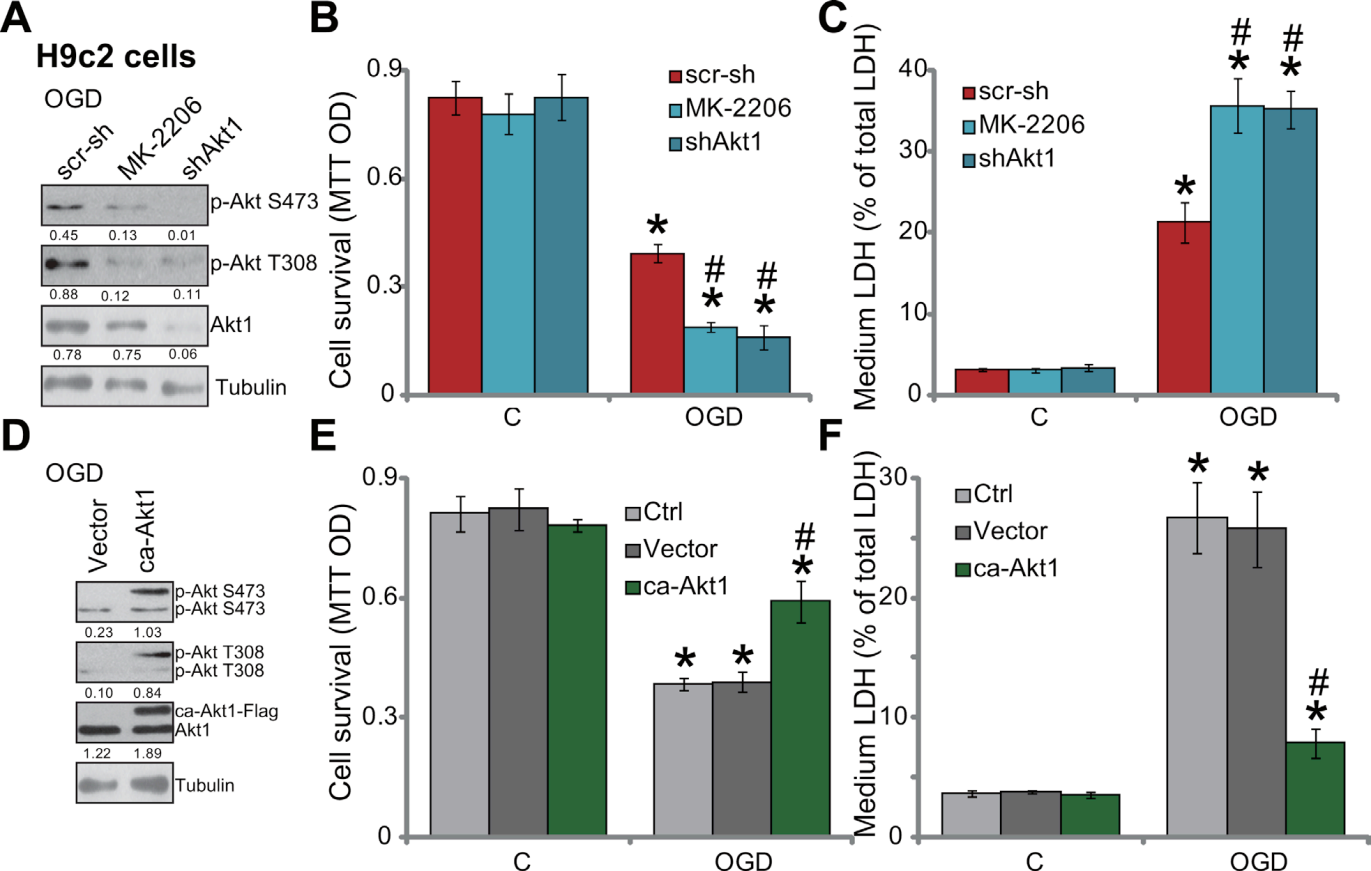
OGD/re-oxygenation-induced myocardial cell death is exacerbated with Akt inhibition, but is attenuated with forced-activation of Akt H9c2 myocardial cells, expressing Akt1 shRNA (“shAkt1”) or scramble shRNA (“scr-sh”), were treated with/out MK-2206 (10 μM), cells were further subjected to OGD/re-oxygenation; Expression of indicated proteins was shown (**A**, 3 hours after re-oxygenation); Cell survival (**B**, 24 hours after re-oxygenation) and cell death (**C**, 24 hours after re-oxygenation) were also tested. H9c2 cells, expressing constitutively active Akt1 (“ca-Akt1”, Flag-tagged) or empty vector (pSuper-puro, “Vector”), were treated with/out OGD/re-oxygenation; Expression of indicated proteins was shown (**D**, 3 hours after re-oxygenation); Cell survival (**E**, 24 hours after re-oxygenation) and cell death (**F**, 24 hours after re-oxygenation) were tested as well. Akt1 expression (vs. Tubulin) and Akt phosphorylation (vs. Tubulin) were quantified (A and D). Bars indicate standard deviation (SD, *n* = 4). Each experiment was repeated three times and similar results were obtained. *p < 0.05 vs. “C” group. #*p* < 0.05 vs. “scr-sh”/“Vector” group.

Based on the results above, we would speculate that Akt inhibition may exacerbate OGD/re-oxygenation-induced myocardial cell death. MK-2206 and Akt1 shRNA were applied again to block Akt activation (p-Akt at Ser-473 and Thr-308) in H9c2 cells with OGD/re-oxygenation (Figure 3A). Significantly, as shown in Figure 3B and 3C, MK-2206 and Akt1 shRNA largely intensified OGD/re-oxygenation-induced H9c2 cell viability reduction (Figure 3B) and cell death (Figure 3C). These results indicate that basal Akt activation is also important for the survival of H9c2 cells under OGD/re-oxygenation.

Next, a constitutively active Akt1 (“ca-Akt1”, Flag-tagged) expression vector [21] was introduced to H9c2 cells (see Method). Western blot assay results in Figure 3D confirmed that ca-Akt1 indeed dramatically provoked Akt activation (p-Akt at Ser-473 and Thr-308) in H9c2 cells. Remarkably, H9c2 cells expressing the ca-Akt1 were resistant to OGD/re-oxygenation (Figure 3E and 3F). Therefore, similar to SC79 co-treatment, forced activation of Akt by expressing ca-Akt1 also inhibited OGD/re-oxygenation damages in myocardial cells.

### Activation of Akt by SC79 inhibits OGD/re-oxygenation-provoked programmed necrosis pathway in myocardial cells

**Figure 4:**
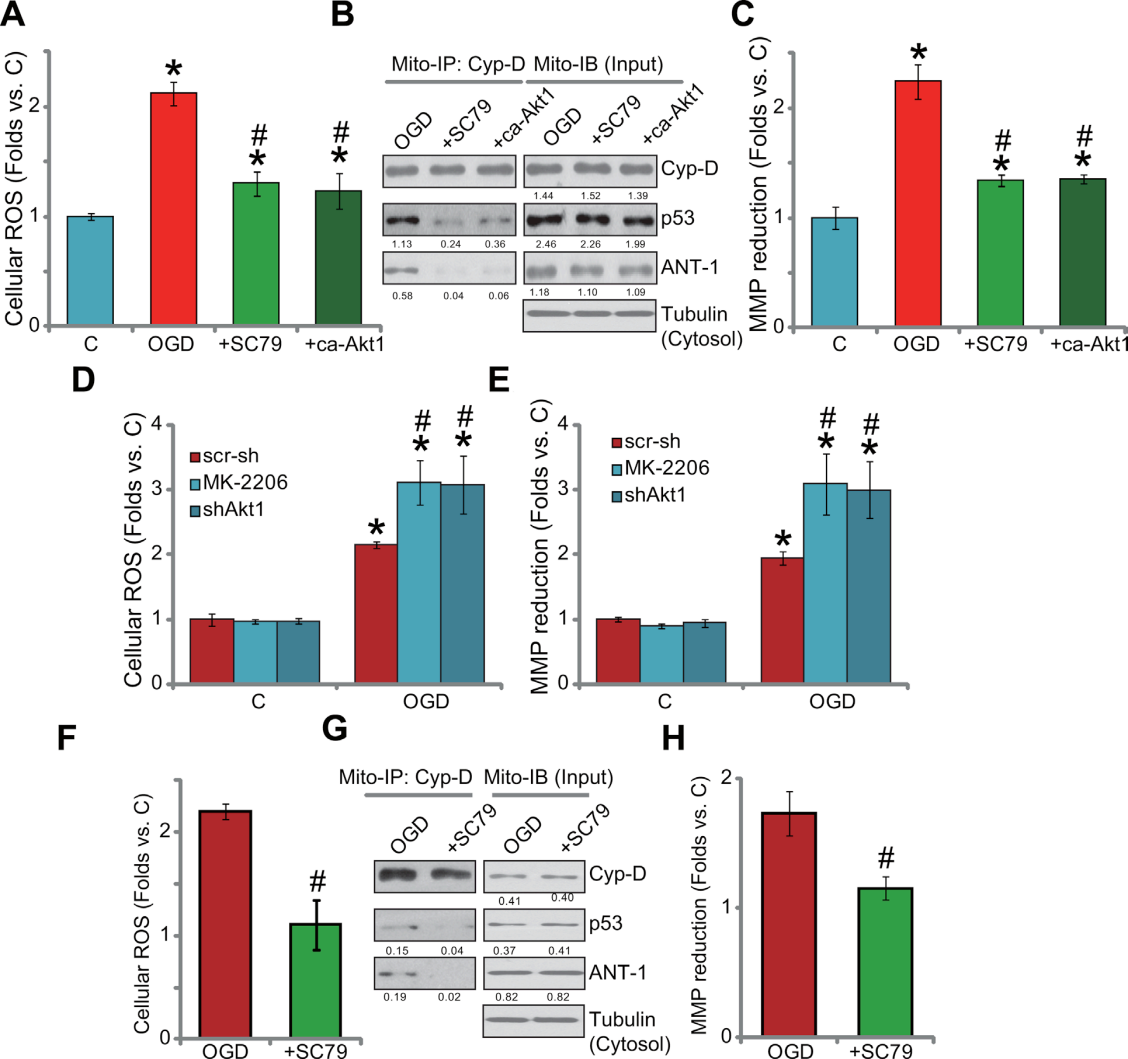
Activation of Akt by SC79 inhibits OGD/re-oxygenation-provoked programmed necrosis pathway in myocardial cells H9c2 cells, with/out constitutively active Akt1 (“ca-Akt1”, Flag-tagged), were treated with OGD/re-oxygenation or plus SC79 (10 μM, 1 hour pre-treatment); ROS production was tested 4 hours after re-oxygenation (**A**); After 6 hours, the complexation and expression of listed proteins in the mitochondrial lysates were tested by mitochondrial immunoprecipitation assay (“Mito-IP” of Cyp-D) and mitochondrial immuno-blot assay (“Mito-IB”, as “Input”), respectively (**B**); The mitochondrial membrane potential (MMP) reduction (JC-10 assay, **C**) was also tested (6 hours after re-oxygenation). The effect of MK-2206 (10 μM, 1 hour pre-treatment) or lentiviral shRNA Akt1 on OGD/re-oxygenation-induced ROS production (**D**, after 4 hours) and mitochondrial depolarization (**E**, after 6 hours) was also tested. Primary murine myocardiocytes were treated with OGD/re-oxygenation or plus SC79 (10 μM, 1 hour pre-treatment), ROS production (**F**, after 4 hours), mitochondrial p53-Cyp-D-ANT-1 complexation and expression (E, after 6 hours) and mitochondrial depolarization (**H**, after 6 hours) were tested using the above methods. For Mito-IP assay, the amount of Cyp-D-bound p53 or ANT-1 was quantified (vs. Cyp-D, B and **G**). For Mito-IB assay, expression of p53/Cyp/D-ANT-1 was quantified (vs. cytosol Tubulin, B and G). Bars indicate standard deviation (SD, *n* = 4). Each experiment was repeated three times and similar results were obtained. *p < 0.05 vs. group “C”. ^#^*p* < 0.05 vs. “OGD” only.

Previous studies have indicated that OGD/re-oxygenation shall activate mPTP (mitochondrial permeability transition pore)-dependent necrosis pathway (but not apoptosis) to mediate myocardial cell death [3, 15]. This mitochondria-mediated cell necrosis pathway is also named programmed necrosis [22–25]. In this study, treatment of OGD/re-oxygenation in the H9c2 cells apparently also provoked this programmed-necrosis pathway, which was evidenced by reactive oxygen species (ROS) production (4A), mitochondrial association of p53-cyclophilin D (Cyp-D)-ANT-1 (adenine nucleotide translocator 1) [26] (Figure 4B) as well as mitochondrial depolarization (Figure 4C). Remarkably, pre-treatment of SC79 as well as expression of ca-Akt1 largely inhibited OGD/re-oxygenation-induced above changes (Figure 4A–4C). On the other hand, blockage of basal Akt activation by MK-2206 or Akt1 shRNA aggravated OGD/re-oxygenation-induced ROS production (Figure 4D) and mitochondrial depolarization (Figure 4E). In the primary murine myocardiocytes, pre-treatment of SC79 similarly inhibited OGD/re-oxygenation-induced ROS production (Figure 4F), mitochondrial p53-Cyp-D-ANT-1 association (Figure 4G) and depolarization (Figure 4H). These results together suggest that activation of Akt by SC79 could inhibit OGD/re-oxygenation-induced programmed necrosis pathway to possibly shut down subsequent myocardial cell death.

### SC79 activates Akt downstream NRF2 signaling in myocardial cells

**Figure 5:**
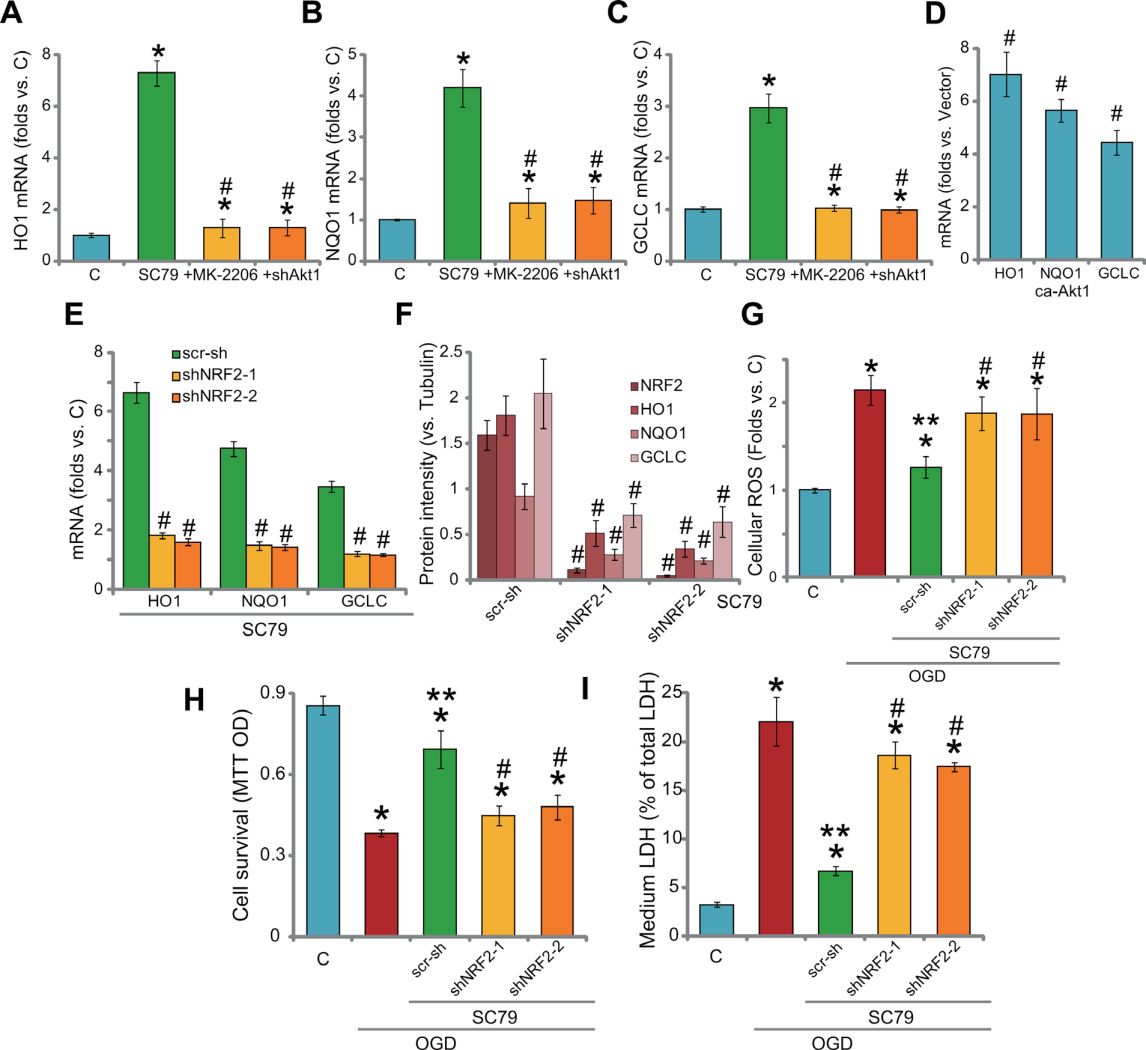
SC79 activates Akt downstream NRF2 signaling in myocardial cells H9c2 myocardial cells, with Akt1 shRNA (“shAkt1”), constitutively active Akt1 (“ca-Akt1”), were treated with/out SC79 (10 μM) or plus MK-2206 (μM) for 2 hours, expression of listed NRF2-regulaed mRNAs was tested by RT-PCR assay (**A**–**D**); H9c2 cells, expressing NRF2 shRNA-1/−2 (“shNRF2-1/2”) or scramble control shRNA (“scr-sh”), were treated with SC79 (10 μM) for 2 hours, mRNA (**E**) and protein (**F**, three repeat data were quantified, vs. Tubulin) expression of listed genes were tested; Above cells were also subjected to OGD/re-oxygenation assay; ROS production (**G**, 4 hours after re-oxygenation), cell survival (**H**, MTT assay, 24 hours after re-oxygenation) and cell death (**I**, LDH release assay, 24 hours after re-oxygenation) were shown. Bars indicate standard deviation (SD, *n* = 3). Each experiment was repeated four times and similar results were obtained. *p < 0.05 vs. “C” group. ^#^p < 0.05 vs. “SC79” group (A-C). ^#^*p* < 0.05 vs. “Vector” cells (D). ^#^*p* < 0.05 vs. “scr-sh” group (D-F).^*^p < 0.05 vs. OGD only group (G-I).

Recent studies have shown that SC79 could activate NRF2 signaling to inhibit oxidative damages [10, 11]. Here, we found that SC79 treatment in H9c2 cells also induced significant mRNA expression of NRF2-regualted genes, including *HO1, NQO1* and *GCLC* (Figure 5A–5C) [10, 11, 27, 28]. Notably, the Akt inhibitor MK-2206 and Akt1 shRNA almost blocked SC79-induced above gene expression (Figure 5A–5C), indicating that Akt serves as the upstream signaling for NRF2 activation by SC79. Notably, exogenous expression of ca-Akt1 in H9c2 cells also induced mRNA expression of above Nrf2 genes (*HO1, NQO1* and *GCLC*, Figure 5D). To confirm that the expression of these mRNAs was through NRF2, shRNA strategy was again applied to silence NRF2. As demonstrated, the two applied shRNAs (see our previous study [3]) dramatically downregulated NRF2 expression in H9c2 cells (Figure 5F). Consequently, SC79-induced mRNA and protein expression of above Nrf2 genes were almost blocked (Figure 5E–5F). These results imply that SC79 activates Akt-dependent NRF2 signaling in H9c2 cells. Significantly, SC79-mediated anti-oxidant activity in OGD/re-oxygenation-treated cells was almost nullified with NRF2 shRNA knockdown (Figure 5G). Further, in the NRF2-silenced cells, SC79-indced cytoprotection against OGD/re-oxygenation was also obviously compromised (Figure 5H and 5I). Therefore, activation of NRF2 by SC79, as a key downstream of Akt, is required for H9c2 cytoprotection against OGD/re-oxygenation.

## DISCUSSION

A number of recent literatures proposed that activation of mitochondrial (programmed) necrosis pathway, but not apoptosis, mediates cell death by sustained OGD/re-oxygenation [3, 15, 29–32]. Following OGD/re-oxygenation, intracellular ROS will be produced, which dictates cytoplasmic p53 translocation to the mitochondria, where it forms a complex with local proteins Cyp-D and ANT-1 [3, 30–32]. The Cyp-D-p53-ANT-1 association is required for subsequent mPTP opening, mitochondrial depolarization and cell necrosis (but not apoptosis) [3, 15, 29–32]. Here, we found that SC79 or ca-Akt1 significantly inhibited OGD/re-oxygenation-induced programmed necrosis pathway, the latter was evidenced by ROS production, mitochondrial depolarization and Cyp-D-p53-ANT-1 mitochondrial association. Thus, we here proposed a novel mechanism of SC79-mediate cytoprotection: To shut down the programmed necrosis pathway.

In the present study, SC79 treatment in myocardial cells also activated NRF2 signaling, as a key downstream of Akt, to block OGD/re-oxygenation-induced ROS production. Reversely, NRF2 shRNA knockdown significantly attenuated SC79-induced ROS scavenging and cytoprotective activities. Thus, activation of NRF2 signaling by SC79 is required for myocardial cytoprotection against OGD/re-oxygenation.

Our results showing SC79 activates NRF2 signaling were consistent with recent findings [10, 11]. Indeed, it has been proposed that Akt and its downstream mTOR complex 1 (mTORC1) could directly activate NRF2 [33, 34]. Lee *et al*., found that sulforaphane provokes NRF2 signaling via PI3K-Akt [35]. Similarly, Xu *et al*., showed that pyocyanin-induced NRF2 activation requires activation of PI3K-Akt [36]. Salvianolic acid A and 3H-1,2-dithiole-3-thione (D3T)-activated NRF2-HO-1 signaling is also the downstream of Akt-mTORC1 signaling [33, 34]. For the mechanism study, it was shown that Akt may induce NRF2 phosphorylation at Ser-40 [10, 11, 33], which is required for subsequent NRF2 stabilization, accumulation and nuclear translocation [33, 37, 38]. Together, we suggest that SC79 activates NRF2 as a downstream of Akt to fight against OGD/re-oxygenation-induced oxidative stress and myocardial cell death. The detailed mechanisms may warrant further studies.

## MATERIALS AND METHODS

### Chemical and reagents

SC79 and MK-2206 were purchased from Selleck (Beijing, China). Antibodies of cyclophilin D (Cyp-D), adenine nucleotide translocator 1 (ANT-1) and p53 as well as NRF2, heme oxygenase-1 (HO1). NADPH quinone oxidoreductase 1 (NQO1) and glutamate cysteine ligase catalytic subunit (GCLC) were obtained from Santa Cruz Biotechnology (Santa Cruz, CA). Antibodies for Akt, phosphorylated (p-) Akt (Thr-308 and Ser-473) and (β-) tubulin were purchased from Cellular Signaling Tech (Beverly, MA).

### H9c2 cell culture

As reported [3, 39, 40], the rat embryonic ventricular H9c2 myocardial cells were cultured in DMEM medium with 10% FBS.

### Primary culture of murine myocardiocytes

Primary culture of murine myocardiocytes was described previously [15]. Briefly, ventricles of C57BL6 mice (at day-1) were minced and digested in 0.5 mg/mL collagenase I (Sigma) for 40 min. The cell suspensions containing primary myocardiocytes were filtered. Cells were then cultured in M-199 medium supplemented with 10% FBS, and plated for 30 min to separate from non-myocardiocytes. The myocardiocytes were then plated in M-199 supplemented with 10% FBS. A confluent monolayer with primary spontaneously beating cells was formed [15]. The protocol of culture of primary cells was approved by the Ethics Committee and IACUC of authors’ institutions.

### OGD/re-oxygenation

The detailed protocol of OGD/re-oxygenation was described previously [3]. Briefly, cells were cultured in a pre-warmed glucose-free balanced salt solution [3]. The solution was then bubbled with an anaerobic gas mix (95% N_2_, 5% CO_2_). Cells were then incubated in the solution for 4 hours to produce oxygen and OGD and then re-oxygenated for 3–24 hours.

### MTT cell survival assay

Cell survival was tested by the 3-[4,5-dimethylthylthiazol-2-yl]-2,5 diphenyltetrazolium bromide (MTT) (Sigma, St. Louis, MO) assay [3, 40].

### LDH detection

Following the applied treatment, cell death was detected by lactate dehydrogenase (LDH) assay using a commercial available LDH kit (Takara, Tokyo, Japan). LDH release (×100 %) was calculated as follows: LDH in conditional medium/(LDH in conditional medium + LDH in cell lysates).

### Western blots

Western blot assay was performed as described previously [3, 40]. Band intensity was quantified and normalized to loading control.

### Mitochondrial immunoprecipitation (Mito-IP)

For each treatment, mitochondria of 10 million H9c2 cells were obtained via “Mitochondria Isolation Kit for Cultured Cells” from Thermo Scientific (Hudson, NH), which was then lysed [3]. Immunoprecipitation (IP) was performed via the anti-Cyp-D antibody (see [30]), and immune complexes were captured with protein G-Sepharose. Afterward, IP samples were subjected to Western blot assay, p53-Cyp-D-ANT-1 association was detected [3, 30].

### Real-time PCR

RNA extraction and reverse transcription were performed as described previously [3] . Real-time PCR was performed on a Bio-Rad IQ5 multicolor detection system. After amplification, melt curve analysis was performed to analyze product melting temperature. *GAPDH* was tested as internal control, and 2^-ΔΔCT^ method [41] was applied to quantify mRNA expression. The primers for rat *HO-1, GAPDH NQO-1* and *GCLC* were described previously [3]. The primers for murine HO-1, GAPDH, GCLC, NQO-1 were from Dr. Jiang's group [33, 34].

### ROS detection

Following treatment of cells, ROS production was determined by carboxy-H2DCFDA dye assay [3]. The ROS intensity was recorded on a spectrofluorometer using excitation and emission filters of 488 and 530 nm, respectively [3].

### Detection of mitochondrial membrane potential (MMP)

MMP was measured through JC-10 dye (Invitrogen) to reflect mitochondrial depolarization [42]. Briefly, after indicated treatment, cells were stained with JC-10 (5.0 μg/mL), which were then washed, tested immediately on a spectrofluorometer.

### NRF2 knockdown by shRNA

The two non-overlapping lentiviral NRF2 shRNAs were described previously [3]. The lentiviral Akt1 (rat) shRNA was purchased from Santa Cruz Biotech. The lentiviral shRNA (20 μL/mL) was added to cells for 12 hours, and cells were selected by puromycin (0.25 μg/mL) for another 24 hours. NRF2 or Akt1 expression in the selected cells was tested by Western blot assay. Control cells were transfected with same amount of scramble control shRNA (Santa Cruz, Shanghai, China).

### Constitutively active Akt1 expression

The construct with constitutively active mutant of Akt1 (“ca-Akt1”, Flag-tagged) as well as empty vector (pSuper-puro) were provided by Jiang's group [21]. The construct was transfected to H9c2 cells via Lipofectamine 2000 (Invitrogen). Cells were then subjected to puromycin (0.25 μg/mL) selection for 48 hours. Expression of Akt1 in the selected cells was tested by Western blot assay.

### Statistical analysis

Statistical significance was determined by one-way ANOVA by Dunnett's test as reported [3, 4, 34].

## CONCLUSIONS

Activation of Akt by SC79 protects myocardial cells from OGD/re-oxygenation damages.
